# Influence of Storing Composite Filling Materials in a Low-pH Artificial Saliva on Their Mechanical Properties—An In Vitro Study

**DOI:** 10.3390/jfb14060328

**Published:** 2023-06-20

**Authors:** Abdulaziz Alhotan, Zbigniew Raszewski, Rasha A. Alamoush, Katarzyna Chojnacka, Marcin Mikulewicz, Julfikar Haider

**Affiliations:** 1 Department of Dental Health, College of Applied Medical Sciences, King Saud University, Riyadh P.O. Box 12372, Saudi Arabia; 2 SpofaDental, Markova 238, 506-01 Jicin, Czech Republic; 3 Department of Prosthodontics, School of Dentistry, The University of Jordan, Amman 11942, Jordan; 4 Department of Advanced Material Technologies, Faculty of Chemistry, Wroclaw University of Science and Technology, Smoluchowskiego 25, 50-372 Wroclaw, Poland; 5 Division of Facial Abnormalities, Department of Dentofacial Orthopaedics and Orthodontics, Wroclaw Medical University, Krakowska 26, 50-425 Wroclaw, Poland; marcin.mikulewicz@umw.edu.pl; 6 Department of Engineering, Manchester Metropolitan University, Manchester M1 5GD, UK

**Keywords:** dental restorative composite, artificial saliva, low pH, flexural strength, crushing resistance

## Abstract

Restorative composites are subjected to various influences in the oral cavity environment, such as high or low temperatures, the mechanical force generated during mastication, colonization of various microorganisms, and low pH, which may result from ingested food and the influence of microbial flora. This study aimed to investigate the effect of a recently developed commercial artificial saliva (pH = 4, highly acidic) on 17 commercially available restorative materials. After polymerization, the samples were stored in an artificial solution for 3 and 60 days and subjected to crushing resistance and flexural strength tests. The surface additions of the materials were examined in terms of the shapes and sizes of the fillers and elemental composition. When stored in an acidic environment, the resistance of the composite materials was reduced by 2–12%. Larger compressive and flexural strength resistance values were observed for composites that could be bonded to microfilled materials (invented before 2000). This may result from the filler structure taking an irregular form, which results in a faster hydrolysis of silane bonds. All composite materials meet the standard requirements when stored for a long period in an acidic environment. However, storage of the materials in an acid environment has a destructive impact on the materials’ properties.

## 1. Introduction

Composite materials are currently the most common fillings used in dentistry. In addition to being used as fillers, they are also used for prosthetics, periodontology, and dental surgery. However, despite this popularity, a large proportion of restorations must be replaced mainly due to secondary caries (47%) followed by restoration fractures (24%) [1]. Over the past 60 years, filler composites have undergone significant changes in filler particles and resins. In the case of resins, new methacrylic resins are used to reduce and counteract stresses that arise during polymerisation shrinkage [2,3]. In the 1970s and 1980s, the main reasons for the failure of composite restorations were attributed to insufficient wear resistance. Improvements in available filler technology have resulted in more durable materials and have changed the causes of failure. The results have been particularly notable when filler particle dimensions or shapes are changed, or reduced fillers are used in the modern composites [4].

Over the last 20 years, a completely new approach has been adopted for the development of filler particles. Instead of grinding glass to smaller sizes, more manufacturers are beginning to produce particles of different filler materials by the hydrolysis of appropriate organic compounds [5,6]. This allows them to immediately obtain particles of the appropriate geometry and uniform size. Furthermore, the energy inputs for grinding materials are greater than those observed for hydrolysis of the relevant compounds [7,8]. Such particles have better polishability and superior optical properties. As such, it is possible to obtain the chameleon effect and adjust the composite material to the colour of the tooth in which it is placed. This facilitates the selection of the colour by the dentist and reduces the number of colours of composite materials used in everyday practise [9]. At present, many composite materials can be found on the market. These composites are manufactured using various technologies, both very modern and those developed in the 1980–90s. The oral environment can have different impacts on different materials [10].

Dental fillings are regularly subjected to a variety of destructive actions. Mechanical degradation can occur through food mastication and opposing teeth coming into contact [11]. In addition, composite materials are also affected by the chemicals present in food, alcohol, dyes, and drinks with an acidic pH level. It is known from the literature that orange, grapefruit, and apple juice, and medium values for Coca-Cola, sparkling orange juice, and white wine have the greatest adverse impact on composite materials. On average, a person consumes about 2–3 L of liquids a day. In developed countries, these liquids typically contain a large amount of sugar, which, after decomposition by bacteria, causes a decrease in the pH level in the oral cavity [12]. The long-term impact of unfavourable chemical factors on restorative composite materials may result from the fact that they are trapped around the margins of inadequately finished restorations, providing conditions for long contact with chemicals [13,14,15,16]. Furthermore, the surface of the teeth and fillings is also colonized by various types of bacteria, the metabolism of which produces acids or low-pH chemicals [17].

In addition to the effect of bacteria, some diseases can cause a decrease in the pH level in the mouth. For example, bulimia, where stomach acid can come into direct contact with teeth due to the forced regurgitation of food. Gastroesophageal reflux disease (GERD), one of the most common digestive tract diseases, can also lead to a decrease in the oral pH level. Prolonged exposure to an acidic environment in the mouth can also cause various diseases; for example, increased susceptibility to caries, periodontal disease, taste disturbances, and halitosis, and an increased incidence of oral infections [18,19]. Different filling materials can also degrade to a greater or lesser extent due to exposure to chemicals with a low pH [19,20]. Several studies have examined the effects of storing composite materials in various media, including artificial saliva, distilled water, alcohol, coffee, and tea [8]. However, most of the existing studies were limited to groups of three to six composite materials. Therefore, a larger group of materials currently available on the market needed to be tested under the same conditions to obtain comparable results for their mechanical properties.

This study aimed to evaluate the effect of a low-pH artificial saliva on the mechanical properties of 17 types of composite materials. The null hypothesis was as follows: there will be no differences in the mechanical properties of the restorative composite materials of different manufacturers after storing them in a low-pH artificial saliva for 3 days and 60 days. In addition, it was decided to investigate the composition of fillers in the composite materials.

## 2. Materials and Methods

### 2.1. Specimen Preparation

A total of 408 samples of composite materials from various manufacturers (Table 1) were cured using a Demi Ultra LED lamp (Kerr, Orange, CA, USA) at 1200 mV/cm^2^. The curing time was 40 s on each side of the sample. Such a long exposure time was chosen to ensure that all samples were properly and uniformly cured. The composite materials can be uncured but not over polymerized according to the principle of double bond reaction [13]. Before the examination, the lamp power was determined using a suitable radiometer. The composites were irradiated with the lamp in direct contact.

The materials, after being removed from each syringe by means of a metal instrument, were applied to the moulds. A detailed sample preparation for flexural and compressive strength is shown in [21].

### 2.2. Mechanical Measurement Procedures

#### 2.2.1. Flexural Strength Measurement

The material extruded from the syringe was placed in a 2 × 2 × 45 mm^3^ unfolded metal mould and covered with PE (polyethylene foil) foil. The material was cured on both sides for 40 s using Demi Ultra (Kerr, Orange, CA, USA) [21]. After curing, the samples were polished and stored in artificial saliva with a Fusayama–Mayer solution (0.4 g/L NaCl, 0.4 g/L KCl, 0.795 g/L CaCl_2_·2H_2_O, 0.005 g/L Na_2_S·9H_2_O, 0.69 g/L NaH_2_PO_4_·2H_2_O, and 1 g/L urea) and 0.4 g HCl at 37 ± 2 °C. All chemicals were purchased from Sigma Aldrich (Praha, Czech Republic) [20].

**Figure 1:**
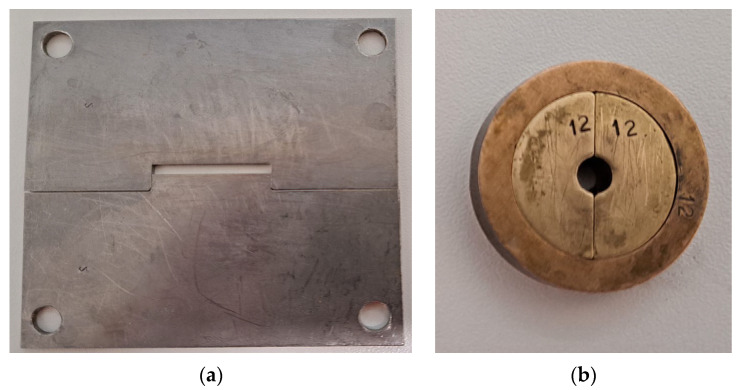
Moulds to prepare samples for (**a**) flexural strength and (**b**) compressive strength.

Twelve samples from each material were prepared for testing (total 204 samples). The first six were fractured by a three-point bending test using a Shimadzu instrument 5 kN (Shimadzu, Kyoto, Japan) after 3 days of storage in artificial saliva with a strong acidic environment (pH = 4) at 37 °C to simulate the oral condition. A support spacing of 40 mm with a load cell capacity of 5 kN and a cross-head travel speed of 5 mm/min were used to fracture the sample. The second group of samples was stored in the same artificial saliva solution for 60 days and then subjected to a breakage test. Equation (1) was used to calculate the flexural strength ($\sigma_{f}$) of each specimen in MPa.

(1) $$\sigma_{f}=\frac{3\mathrm{FL}}{2{\mathrm{bh}}^{2}}$$

where F is the maximum load applied to the specimen in N, L is the length of the support span (40 mm), b is the width of the specimen in mm, and h is the thickness of the specimen in mm. A detailed description of this test is included in ISO 4049:2019—Polymer-based restorative materials. The flexural strength sample preparation tool is shown in Figure 1a.

#### 2.2.2. Compressive Strength Measurement

Crush resistance is not a test required by the ISO 4049 standard [21], however, it provides valuable information on how the material will perform in Class 2 cavities in the molars. As such, the same group of composite materials was also crush tested. The samples were placed in unfolded metal moulds of a diameter of 4.5 mm and a height of 12 mm. These moulds were then covered with PE foil on both sides. The material was cured on both sides for 40 s using Demi Ultra (Kerr, Orange, CA, USA) [21].

The process employed in this study was described in detail by Al-Shekhli et al. [22]. Specifically, 12 samples of each composite material were made (204 total). The first group was subjected to a compression test after 3 days of storage in an artificial saliva at pH = 4, and the second group after 60 days. Shimadzu equipment (Shimadzu, Kyoto, Japan) was used for the tests, and the crushing speed was 0.6 mm/min. The test ended when the sample was deformed (Equation (2)).

(2) $$P=F/\pi r^{2}$$

where *F* is the maximum load at the breaking point and *r* is the diameter of the sample.

The compressive strength sample preparation mould is shown in Figure 1b.

### 2.3. Fracture Surface Analysis

For the SEM analysis, the surfaces of the broken samples from the flexural strength test were used after 60 days of storage in an artificial saliva with pH = 4. A scanning electron microscope (SEM) (Carl Zeiss Ltd., 40 VP, Smart SEM, Cambridge, UK) was employed to study the fractured surface of the tested group of specimens, while energy dispersive X-ray spectroscopy (EDX) analysis was also employed to display the elements inside all the tested composite materials. The samples were randomly selected and loaded into SEM/EDX to be imaged by a secondary electron detector with an acceleration voltage of 20.0 kV. The same technology was used to determine the level of adherence between the fillers and the resin matrix, the existence of any defects, and the sample porosity. The specimens were mounted on aluminium stubs before being sputter coated with a wafer-thin gold layer.

### 2.4. Statistical Analysis

Statistical software (SPSS statistics version 27, IBM, New York, NY, USA) was employed to analyse the compressive and flexural strength data. A Levene test was performed, and the resulting *p*-values indicated that the data exhibited normal distribution and homogeneous variance. To investigate any variations in the flexural and compressive strengths of the materials over time, a one-way analysis of variance (ANOVA) was performed using a Tukey post hoc test, with a significance threshold of *p* ≤ 0.05. Furthermore, to examine the effect of the acidic environment on the materials, an independent t-test was used to calculate the difference between samples stored for 3 and 60 days. The obtained *p*-value was also set at a significance level of *p* ≤ 0.05.

## 3. Results

### 3.1. Flexural Strength

The results of the flexural strength tests with one-way ANOVA are presented in Table 2.

The ANOVA results revealed that there was a significant difference (*p* ≤ 0.05) in the flexural and compressive strengths of the materials stored for 3 and 60 days. However, for several materials, there was no significant variation (*p* > 0.05) between storage periods in flexural strength and compressive strength. On the other hand, the t-test demonstrated that some materials had significant differences across storage lengths (3 d and 60 d comparison). However, it should be noted that some materials did not exhibit any significant differences in terms of storage duration.

In terms of flexural strength, the TPH Spectrum (Dentsply) composite exhibited the highest strength (176 MPa and 163 MPa) after both the 3-day and 60-day storage durations, whereas the Premise (Kerr) composite had the lowest flexural strength following both storage periods. After storage in the acidic medium, the flexural strength of the composite materials was reduced by 2–16%. The largest differences were observed for the Majesty Flow material (16%) and Premise (11%).

### 3.2. Compressive Strength

Table 3 shows the results of the compressive strength tests after the samples were stored in an artificial saliva (pH = 4) for 3 and 60 days.

For the compressive strength test, the Majesty Flow (Kuraray) composite had the highest values (365 MPa and 356 MPa) after the 3- and 60-day storage durations, while the Point 4 (Kerr) composite showed the lowest compressive strength after both storage periods. The low pH also caused changes in the compressive strength of the materials, which, after storage at a low pH, had lower values ranging from 2–18%. The greatest difference was observed for the Herculite Flow material (from 358.52 ± 26.6 MPa to 290.36 ± 10.6 MPa after 60 days). A greater reduction in this parameter was seen for older generation materials. For this group of materials, greater separation between resistances was observed for materials with viscosities higher than those of the flow-type materials. For more contemporary materials, the flowable composites had the same mechanical properties as the packable materials.

### 3.3. Microstructural Characteristics

**Figure 2:**
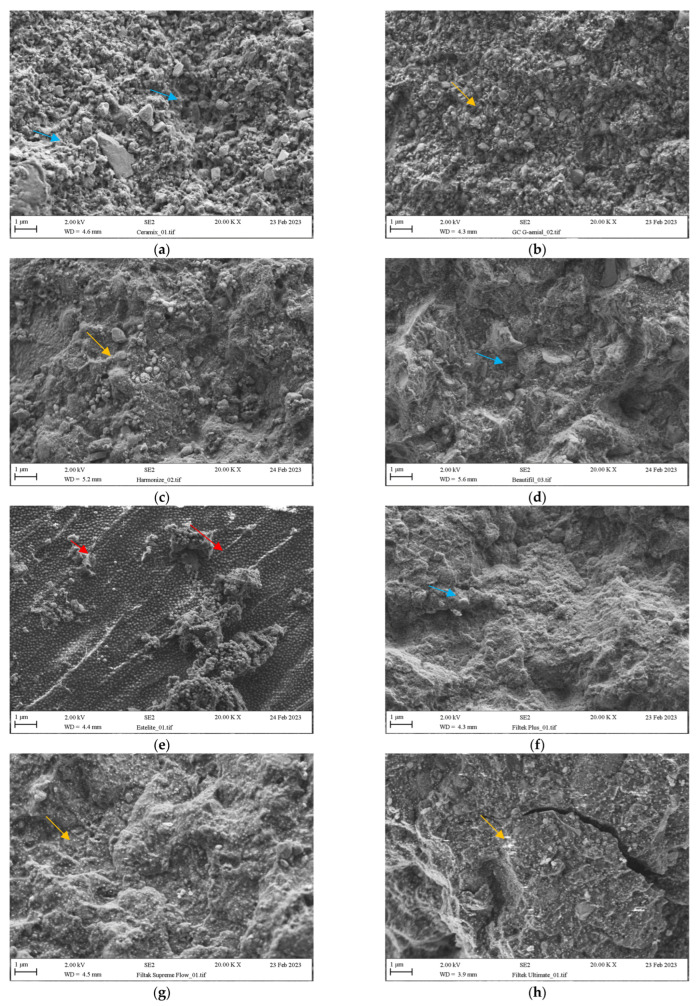
(**a**) Ceramix (**b**) G-aenial (**c**) Harmonize (**d**) Beatyfil (**e**) Estelite (**f**) Filtek Plus (**g**) Filtek Supreme Flow (**h**) Filtek Ultimat. Blue arrows indicate irregular small and big particles, red arrows uniform spheres, and yellow arrows spherical particles.

The SEM analysis results presented in Figure 2a–g indicate two types of fillers used in composites: mechanically ground materials visible in the form of irregular shapes and a spherical filler in Estelite (Figure 2d). Table 4 presents further information on the types of filler.

The fractured surface varies between the materials tested. In the case of Filtek Ultimate, crack lines are visible inside the structure of the material and the surface is smooth, as if the crack occurred at the border of the organic phase. No filler is visible. Another case is the Estelite material, where crack lines are visible on the surface. Similarly, the crack limit runs on the surface of the organic phase for Beatyfill and Filtek Plus.

### 3.4. Chemical Characteristics

The obtained elemental compositions are also shown in weight percentages in Table 5.

The composition of the composite materials includes the following elements: carbon, oxygen, aluminium, and silica. Natrium is missing in Filtek Ultimate and Tetric Ceram, while the remaining materials contain <1% Na. The exceptions in terms of the missing sodium atoms inside the glass were G-aenial and Beatifil, which are both from Japan. Zirconium ions were detected in Filteck, Tertic Ceram, Ominchroma, Estelite, and Harmonize, all of which were introduced to the market after 2000. The fluorine content ranges from 2–8%, but it is not present in all materials, which in small amounts may be secreted into the oral cavity [23]. The carbon content indicates the amount of organic phase in the material, which is greater in the case of flow materials. Strontium, barium, and zirconium are required for the restoration to be X-ray visible.

## 4. Discussion

The findings of this study confirm that there are significant differences between the flexural strength and the compressive strength of the tested materials. Thus, the null hypothesis has to be rejected. Storing the samples in a low-pH solution decreases their resistance to bending, which is consistent with the results obtained by other authors. Flexural strength allows composites to withstand masticatory loads in a clinical situation. As such, it is one of the most important mechanical properties tested because it directly influences the lifetime of restoration in the oral cavity [13]. On average, these values were approximately 10% lower after 2 months of storage than after 3 days of storage. Large changes in the reduction of mechanical resistance to fracture can be observed in the case of materials with large regular filler particles, e.g., Herculite Flow (compressive strength 358.52 ± 26.6 MPa after 3 days and 290.36 ± 10.6 MPa after 60 days). Similarly, for the same material with the same ageing period, the flexural strength was reduced from 144.54 ±11 MPa to 134.68± 5.9 MPa. This may be due to the fact that chemical hydrolysis causes weakening and leaching of this type of molecule, which results in larger defects in the surface of the material and reduces the mechanical resistance. This process can be further accelerated by the rapid leaching of sodium ions from the material (content of 0.4% in Herculite). For comparison, the Filtek Flow material does not contain sodium ions, and the SEM images indicate that it has smaller and more regular filler particles. After prolonged storage in an environment of pH = 4, the flexural strength was 145.56 ± 5.8 MPa after 3 days and 143.16 ± 9.6 MPa after 60 days. A similarly smaller weakening can be observed for the compressive strength of the same material (280.70 ± 22.1 MPa after 3 days and 266.86 ± 18.1 MPa after 60 days).

The reduction of mechanical properties could be attributed to the fact that the composite resin fillers can be leached out of the material, and the matrix component decomposes under the influence of a low-pH environment [23]. The second explanation for this phenomenon is that unpolymerized methacrylate monomers are leached out of the interior of the materials. The water begins to penetrate, which has a plasticising effect on the composite material [24]. However, these results were not observed for all materials. The majority of the materials, according to the manufacturers’ recommendations, require a polymerization time of 10 or 20 s, while Premise and G-aenial Universal require 30 and 40 s, respectively, which may result in a different way of curing the materials after the 40 s used in this study. When the materials were polymerized for a long time in these tests, a higher degree of conversion of double bonds in the methacrylic resins can be obtained [7]. This is consistent with the results obtained by Scribante et al. for a similar group of composite materials tested in acidic beverages (Coca-Cola) [13].

In the literature, citric acid has been used as an example of a low-pH environment. Some authors have found that 2% citric acid is not destructive to some composite materials. The exposure time of a given medium has a great influence on the hydrolysis. This process begins with the gradual decomposition of the weakest element in the composite material, a layer of silane on the surface of the filler. In the next step, the filler particle is released [25,26]. Furthermore, the results obtained in this study are in agreement with those of Mckinney et al., who concluded that weak organic acids, such as citric and lactic acids, have a damaging effect on inorganic fillers and, for this reason, can influence the reduction of the flexural strength of the composite [27]. In this case, it is artificial saliva, which contains a small amount of a stronger acid than lactic or citric acid, such as HCl.

The minimal flexural strength required by the ISO standard for composite materials used for occlusal filling is at least 80 MPa after 24 h of storage in distilled water [21]. Our tests show that all materials will meet this requirement within 60 days in an acidic environment, that is, in conditions more severe than the normative requirements. In addition to affecting the flexural strength of the composite materials (packable and flow type), the filler content also plays a relevant role in the modification of the flexural strength value [28], which has been confirmed in these studies. For example, material from the same manufacturer but with different amounts of the same filler have different mechanical properties (the compressive strengths of Filtek Supreme (3M) were 361.76 ± 37.0 MPa and 299.66 ± 12.9 MPa after 3 days and 60 days while Filtek Flow (3M) displayed compressive strengths of 280.70 ± 22.1 MPa after 3 days and 266.86 ± 18.1 MPa after 60 days).

The content of the organic phase also affects the mechanical properties of the material, which can be observed for the TPH Spectrum (Dentsply) and TPH Spectrum Flow (Dentsply) materials where the packable material has a carbon content of 33.89 ± 0.94% and the Flow material has 43.62 ± 0.50%.

The amount of silanes used in relation to the size of the filler particles can also affect how the fracture progresses within the material. At the initial stage, the fracture of brittle materials occurs through the propagation of pre-existing cracks under the influence of tensile stresses. These cracks may be formed due to the inclusion of defects/blow holes during material processing, polishing (deep micro-scratches), or imperfections in the microstructure [29]. As reported by some authors, mechanical resistance may also be influenced by the triethylene glycol dimethacrylate (TEGDMA) contained in the monomer, leading to increased water uptake [4]. Smooth spherical filler particles are associated with an increased filler volume fraction due to improved particle packing and greater fracture toughness, which can be observed in some materials, for example, Estelite (Figure 2e). The carbon content indicates the amount of organic phase in the products. The lower the concentration of this element, the higher the percentage of filler. For some materials, such as Filtek and TPH Flow, the C content is more than 40%, while others, such as Point 4 and Gaenial, contain less than 30%. In these cases, the content of methacrylic resins is much lower (Tetric Ceram 26.01 ± 1.77% C; Omnichroma 27.75 ± 0.66% C). The presence of Sr, Ba, and F indicates that the material contains glass in its composition, which involves high-temperature melting and grinding to small particles (Beautifil, G-aenial, G-aenial Flow, Ceramix). The presence of Zr indicates that the filler was made by hydrolysis of appropriate organic compounds containing silicon and zirconium (Filtek Ultimate, Omnichroma, Harmonize).

According to numerous authors, the lower the pH value, the faster the erosion of composite materials. Some drinks, such as orange juice or Coca-Cola, have a pH of 3 [15,30]. In this case, the artificial saliva was a buffered solution with a constant pH of 4; therefore, it showed lower erosive properties compared to the materials tested. In addition to the factors listed above, various diseases also cause a decrease in pH in the oral cavity environment, including acid reflux [31]. In terms of compressive strength, flowable materials with less filler have lower values. This is because the resin matrix is more plastic and less resistant to compressive forces [32]. Some authors have observed lower crush resistance values when samples are stored in a low-pH artificial saliva. This type of test can also help determine changes inside composite materials subjected to the action of various factors [33].

Materials containing smaller particles below 1 µm show greater resistance to fracture and crushing. The flexural resistance results obtained in our tests are comparable to those declared by the material manufacturers on the internet and in work by Scribante at al [13]. However, the normative values are obtained with materials stored for 24 h in distilled water and not all products have flexural strength (FS) values available from the producers.

## 5. Conclusions

- The hypothesis assumed for this study has been rejected as significant differences in mechanical properties between different composites were found when aged in a low-pH artificial saliva.
- The flexural resistance of the composite materials after 60 days of storage in a pH = 4 environment was reduced by 2–16%, depending on the product.
- The same phenomena could be observed for the compressive strength values of the materials. Specifically, after 60 days of storage in a low-pH medium, the strength of the materials were between 5 and 20% weaker.
- Materials in their composition contain various filler particles. Some of them have a filler of 1 um. The particles may be irregular or spherical.
- Large and irregular particles are obtained by grinding molten glass. Smaller spherical filler particles are generated by the hydrolysis of appropriate organic compounds such as tera ethoxy, silane, and zirconium organic compounds.
- Some materials contain fluoride, which can be released in small amounts from excision and is an element that prevents caries.

## Figures and Tables

**Table 1:** Composite materials used for testing, according to the information provided by manufacturers.

Material	Producer	Main Filler Composition	Curing Condition Recommended by the Manufacturer (Maximal Thickness of Cure and Time)
Premise	Kerr (Orange, CA, USA)	Prepolymerised filler, barium glass, silica nanoparticles, acrylic resins, photo initiator	2.5 mm, 40 s
Point 4	Kerr (Orange, CA, USA)	Barium aluminoborosilicate, acrylic resins, photo initiator	2–3 mm, 20 s
Filtek Supreme Plus	3M/ESPE (St. Paul, MN, USA)	76.5% SiO_2_ nanosilica filler, ZrO_2_/SiO_2_ nanoclusters, acrylic resins, photo initiator	2 mm, 20 s
Gradia Direct	GC America (Alsip, IL, USA)	Silica, prepolymerized fillers, fluoro-alumino-silicate glass, acrylic resins, photo initiator	3 mm, 10 s
Tetric Ceram	Ivoclar (Schaan, Lichtenstein)	Barium glass, ytterbium triflouride, Ba–Al floursilicate, silicon dioxide, acrylic resins, photo initiator	3 mm, 10 s
Estelite	Tokuyama (Yamaguchi ken, Japan)	82% spherical silica, acrylic resins, photo initiator	3 mm, 10 s
Omnichroma	Tokuymama (Yamaguchi ken, Japan)	Spherical silica, acrylic resins, photo initiator	3.5 mm, 20 s
Filtek Supreme Flow	3M/ESPE (St. Paul, MN, USA)	SiO_2_ nanosilica filler, ZrO_2_/SiO_2_ nanoclusters, YtF_3_ acrylic resins, photo initiator	2 mm, 20 s
Herculite Flow	Kerr (Orange, CA, USA)	Barium glass filler (0.4 µm), silicon dioxide (0.02–0.05 µm), acrylic resins, photo initiator	2 mm, 20 s
G-aenial Universal	GC America (Alsip, IL, USA)	76% silicon dioxide, strontium glass (10–200 nm), acrylic resins, photo initiator	2 mm, 30 s
Beautifil	Shofu (Kyoto, Japan)	S-PRG filler based on fluoroboroaluminosilicate glass, acrylic resins, photo initiator	4 mm, 20 s
TPH Spectrum	Dentsply (Charlotte, NC, USA)	76% blend of spherical, pre-polymerized SphereTEC fillers (d3, 50 ≈ 15 µm), non-agglomerated barium glass and ytterbium fluoride, acrylic resins, photo initiator	2 mm, 20 s
G-aenial Flow	GC America (Alsip, IL, USA)	Silicon dioxide, strontium glass (10–200 nm), acrylic resins, photo initiator	2 mm, 10 s
Ceramix	Dentsply (Charlotte, NC, USA)	76% SphereTEC^®^ filler technology, acrylic resins, photo initiator	2–3 mm, 20 s
Harmonize	Kerr (Orange, CA, USA)	Nanoparticle filler ZrO_2_ SiO_2_, acrylic resins, photo initiator	1–3 mm, 10 s
TPH Spectrum Flow	Dentsply (Charlotte, NC, USA)	SphereTEC™ technology ZrO_2_ SiO_2_, acrylic resins, photo initiator	2 mm, 20 s
Clearfil Majesty Flow	Kuraray America (New York, NY, USA)	Pre-polymerized filler, silanased barium glass, acrylic resins, photo initiator	3 mm, 10 s

**Table 2:** Flexural strength of the tested materials after being stored in an artificial saliva solution of pH = 4 for 3 and 60 days.

Material	Flexural Strength after 3 Days (MPa)	Flexural Strength after 60 Days (MPa)
Ceramix (Dentsply)	138.86 ± 6.5 ^ABCD^	136.80 ± 12.3 ^BCD^
Tetric Evo Ceram (Ivoclar)	133.86 ± 3.9 ^ABC^	132.50 ± 8.3 ^BCD^
Beautifil (Shofu)	132.00 ± 6.5 ^ABC^	130.66 ± 5.8 ^BCD^
Omichroma (Tokuyama)	133.12 ± 5.6 ^ABa^	128.30 ± 7.8 ^BCDb^
Premise (Kerr)	125.42 ± 6.4 ^Aa^	111.34 ± 5.6 ^Ab^
G-aenial (GC)	136.10 ± 7.8 ^ABCDa^	123.98 ± 8.0 ^ABb^
Point 4 (Kerr)	143.91 ± 8.3 ^ABCDE^	142.86 ± 13.0 ^CD^
Harmonize (Kerr)	148.32 ± 11.7 ^BCDE^	139.20 ± 6.6 ^BCD^
Estelite (Tokuyama)	126.55 ± 6.7 ^A^	124.74 ± 7.4 ^ABC^
TPH Spectrum (Dentsply)	176.92 ± 10.0 ^Fa^	163.68 ± 8.4 ^Eb^
Geanial Flow (GC)	150.12 ± 9.1 ^CDE^	148.60 ± 9.3 ^DE^
Majesty Flow (Kuraray)	163.18 ± 12.5 ^EFa^	137.96 ± 10.1 ^BCDb^
TPH Spectrum Flow (Dentsply)	151.42 ± 7.3 ^CDEa^	134.76 ± 7.3 ^BCDb^
Filtek Supreme (3M)	148.68 ± 9.3 ^BCDE^	145.56 ± 5.8 ^DE^
Herkulite Flow (Kerr)	144.54 ± 11.0 ^ABCDE^	134.68 ± 5.9 ^BCD^
Filtek Supreme Flow (3M)	154.44 ± 9.7 ^DE^	148.58 ± 10.0 ^DE^
Filtek Flow (3M)	145.56 ± 5.8 ^ABCDE^	143.16 ± 9.6 ^CD^

Note: In each column, similar uppercase superscript letters indicate that there are no significant differences between materials (*p* > 0.05). In each row, different lowercase superscript letters indicate significant differences between exposure time (3 d and 60 d) within a material (*p* ≤ 0.05).

**Table 3:** Compressive strength of the materials tested after being stored in an artificial saliva solution of pH = 4 for 3 and 60 days.

Material	Compressive Strength after 3 Days (MPa)	Compressive Strength after 60 Days (MPa)
Ceramix (Dentsply)	353.04 ± 25.5 ^Ca^	320.1 ± 19.6 ^CDEFGb^
Tetric Evo Ceram (Ivoclar)	331.18 ± 14.9 ^BC^	310.76 ± 18.2 ^CDE^
Beautifil (Shofu)	236.62 ± 12.6 ^BCa^	323.44 ± 32.4 ^CDEFGb^
Omichroma (Tokuyama)	341.22 ± 29.0 ^C^	340.68 ± 23.8 ^EFG^
Premise (Kerr)	343.88 ± 21.7 ^C^	327.0 ± 29.5 ^DEFG^
G-aenial (GC)	351.4 ± 8.5 ^C^	348.20 ± 12.5 ^EFG^
Point 4 (Kerr)	261.60 ± 14.9 ^A^	245.22 ± 20.9 ^EFG^
Harmonize (Kerr)	350.80 ± 28.3 ^C^	334.72 ± 14.1 ^DEFG^
Estelite (Tokuyama)	350.8 ± 28.4 ^C^	340.8 ± 15.7 ^EFG^
TPH Spectrum (Dentsply)	351.3 ± 23.6 ^C^	342.84 ± 31.2 ^EFG^
Geanial Flow (GC)	334.94 ± 14.7 ^BC^	332.27 ± 18.6 ^DEFG^
Majesty Flow (Kuraray)	365.02 ± 36.3 ^C^	356.84 ± 20.6 ^G^
TPH Spectrum Flow (Dentsply)	316.56 ± 8.8 ^ABC^	313.11 ± 7.9 ^CDEF^
Filtek Supreme (3M)	361.76 ± 37.0 ^Ca^	299.66 ± 12.9 ^BCDb^
Herkulite Flow (Kerr)	358.52 ± 26.6 ^Ca^	290.36 ± 10.6 ^BCb^
Filtek Supreme Flow (3M)	357.62 ± 14.0 ^C^	349.04 ± 28.7 ^EFG^
Filtek Flow (3M)	280.70 ± 22.1 ^AB^	266.86 ± 18.1 ^AB^

Note: In each column, similar superscript uppercase letters indicate no significant differences between materials (*p* > 0.05). In each row, different lowercase superscript letters indicate significant differences between exposure time (3 d and 60 d) within a material (*p* ≤ 0.05).

**Table 4:** Microstructural characteristics of all resin materials.

Material	Type of Fillers Detected by SEM Photos
Ceramix (Dentsply)	Irregular, small, and large particles from 1–5 µm
Tetric Evo Ceram (Ivoclar)	Spherical 200–500 nm, irregular 1 µm
Beautifil (Shofu)	Irregular shape 1 µm
Omichroma (Tokuyama)	Uniform spheres with a diameter of 200–300 nm
Premise (Kerr)	Irregular 1–5 µm
G-aenial (GC)	Spherical 0.5–1 µm
Point 4 (Kerr)	irregular 1–5 µm
Harmonize (Kerr)	Spherical 200–500 nm, irregular 1 µm
Estelite (Tokuyama)	Uniform spheres with a diameter of 200–300 nm
TPH Spectrum (Dentsply)	Irregular, small, and large particles from 1–5 µm
G-aenial Flow (GC)	Spherical 0.5–1 µm
Majesty Flow (Kuraray)	Spherical 0.5–1 µm
TPH Spectrum Flow (Dentsply)	Spherical 0.5–1 µm
Filtek Supreme (3M)	Spherical 0.5–1 µm
Herkulite Flow (Kerr)	Irregular, small and big particles from 1–5 µm
Filtek Supreme Flow (3M)	Spherical irregular 0.5–1 µm
Filtek Flow (3M)	Spherical regular 100–200 nm bigger irregular 0.5–1 µm

**Table 5:** Elemental composition (wt.%) of individual materials obtained by EDX analysis.

Material	C	O	Na	Al	Si	Zr	Sr	Ba	F
Majesty Flow	34.98 ± 1.76	52.54 ± 2.83	0.51 ± 0.09	2.11 ± 0.09	8.02 ± 0.79	-	-	1.84 ± 0.58	-
G-aenial	29.81 ± 0.46	52.47 ± 0.84	0.21 ± 0.08	2.59 ± 0.37	9.33 ± 0.76	-	-	2.13 ± 0.74	3.44 ± 0.34
Filtek Ultimate	43.09 ± 0.3	47.12 ± 0.65	-	1.39 ± 0.09	6.59 ± 0.67	1.80 ± 0.31		-	-
TPH Flow	43.62 ± 0.50	45.49 ± 0.58	-	1.79 ± 0.21	4.85 ± 1.07	-	-	-	4.13 ± 0.25
Herculite	36.35 ± 0.92	48.81 ± 0.21	0.40 ± 0.01	2.91 ± 0.05	5.48 ± 0.64	-	-	-	6.04 ± 0.06
Ceramix	30.11 ± 3.37	49.65 ± 3.6	0.53 ± 0.19	2.72 ± 0.45	10.15 ± 3.06	-	-	1.45 ± 0.35	3.04 ± 0.49
Premise	27.48 ± 1.19	54.35 ± 2.36	0.72 ± 0.07	2.67 ± 0.09	12.72 ± 1.8	-	-	2.04 ± 1.07	
TPH	33.89 ± 0.94	50.78 ± 0.41	-	2.39 ± 0.03	8.02 ± 0.62	-	-	1.74 ± 0.7	3.17 ± 0.1
Beautifil	29.51 ± 0.52	41.81 ± 0.98	1.39 ± 0.0	6.48 ± 0.44	5.05 ± 0.51	-	7.71 ± 0.74		8.04 ± 0.26
Tetric Ceram	26.01 ± 1.77	47.74 ± 1.9	-	4.17 ± 0.45	13.32 ± 1.82	1.13 ± 0.23	-	3.19 ± 1.25	4.41 ± 0.12
Omnichroma	27.75 ± 0.66	54.18 ± 0.43	0.95 ± 0.06	0.64 ± 0.1	13.28 ± 0.72	3.18 ± 0.34	-	-	-
Harmonize	32.13 ± 1.48	49.26 ± 1.19	0.72 ± 0.03	2.00 ± 0.05	9.85 ± 2.37	2.26 ± 0.56	-	-	3.75 ± 0.26
Point 4	27.67 ± 0.53	57.26 ± 1.35	0.46 ± 0.03	2.82 ± 0.01	10.34 ± 0.44	-	-	1.43 ± 0.36	
G-aenial Flow	27.71 ± 0.58	41.12 ± 1.01	1.49 ± 0.09	6.79 ± 0.29	5.72 ± 0.63	-	8.87 ± 0.99	-	8.27 ± 0.38
Estetlite	39.25 ± 1.06	54.46 ± 1.05	0.60 ± 0.1	2.62 ± 0.98	2.51 ± 0.64	0.53 ± 0.19	-	-	-
Filtek Plus	26.07 ± 1.15	50.58 ± 3.25	0.27 ± 0.1	0.34 ± 0.07	15.33 ± 2.81	7.39 ± 1.55	-	-	-
Filtek Supreme	42.90 ± 0.57	48.44 ± 0.48	0.36 ± 0.13	1.26 ± 0.17	5.49 ± 0.74	1.52 ± 0.25	-	-	-

## Data Availability

The data presented in this study are available within the article.
